# Sequence characterization of a Peruvian isolate of *Sweet potato chlorotic stunt virus*: Further variability and a model for *p22* acquisition

**DOI:** 10.1016/j.virusres.2011.01.010

**Published:** 2011-04

**Authors:** Wilmer J. Cuellar, Regina K. Cruzado, Segundo Fuentes, Milton Untiveros, Maria Soto, Jan F. Kreuze

**Affiliations:** a Virology Laboratory, International Potato Center (CIP), Lima, Peru; b Applied Biotechnology Laboratory, International Potato Center (CIP), Lima, Peru; c Donald Danforth Plant Science Center, Saint Louis, MO 63132, USA

**Keywords:** Crinivirus, SPCSV, p22, Sweetpotato, Evolution

## Abstract

*Sweet potato chlorotic stunt virus* (SPCSV) is probably the most important virus infecting sweetpotato worldwide, causing severe synergistic disease complexes with several co-infecting viruses. To date only one isolate (Ug), corresponding to the EA strain has been completely sequenced. It was later shown to be unusual in that, in contrast to most isolates, it encoded an additional p22 protein at the 3′ end of RNA1. We report the complete sequence and genome organization of a Peruvian isolate of SPCSV (m2-47) as determined by siRNA deep sequencing. We confirm that the ORF encoding p22 is lacking from m2-47 and all tested Peruvian and South American isolates, whereas additional isolates containing *p22* were identified from Uganda. Other potentially important genomic differences such as two small ORFs encoding putative small hydrophobic proteins instead of one, upstream the *hsp70h* gene and a more divergent sequence at its RNA1 3′-UTR in contrast to SPCSV isolates that contain *p22* are discussed and a model for recent acquisition of *p22* in Uganda is proposed. A role for *p22* as a pathogenicity enhancer of SPCSV is also provided by complementary expression of *p22* in transgenic sweetpotato plants.

*Sweet potato chlorotic stunt virus* (SPCSV) is a bipartite member of the family *Closteroviridae,* genus *Crinivirus*. It is phloem-limited, whitefly transmitted and among the largest of the single-stranded positive-sense RNA viruses with a total genome size of ∼17.6 kbp (Dolja et al., 2006; Kreuze et al., 2002). SPCSV has a worldwide distribution including the main sweetpotato production areas of the crop in Africa and the Americas (Gibson et al., 1998; Gutierrez et al., 2003; Kokkinos and Clark, 2006; Valverde and Moreira, 2004) and severe diseases have been associated with mixed infections with SPCSV in Africa (Gibson et al., 1998; Mukasa et al., 2006), Israel (Milgram et al., 1996), Central America (Valverde and Moreira, 2004) and South America (Di Feo et al., 2000; Gutierrez et al., 2003). Experimentally these mixed viral infections are characterized by an accumulation of only the co-infecting virus or viruses. The most dramatic example is the disease caused by the interaction of SPCSV with the potyvirus *Sweet potato feathery mottle virus* (SPFMV; Potyvirus) which causes ‘sweetpotato virus disease’ (SPVD), the economically most important disease of this crop, associated with yield losses of >60% (Gibson et al., 1998; Gutierrez et al., 2003; Untiveros et al., 2007). SPCSV can be distinguished into two distantly related strains EA and WA based on serology and nucleotide sequence data (Hoyer et al., 1996b, Vetten et al., 1996; Tairo et al., 2005). Only one complete genome of SPCSV has been sequenced so far, corresponding to isolate Ug of the EA strain (Kreuze et al., 2002).

Interestingly, partial sequence analyses of other strains suggest that the genome of Ug isolate is unusual in that it contains a *p22* ORF at the 3′ end of RNA1 (Cuellar et al., 2008). Isolates showing a similar genome organization to Ug, which we hereby designate strain EA^p22^, have so far been reported only from Uganda while isolates lacking *p22* and corresponding to both EA and WA strains have been reported in Peru, Israel, and Tanzania, suggesting that the latter is more widely distributed (Cuellar et al., 2008). Another unusual feature of the Ug isolate is that, unlike other criniviruses, the 3′-UTR of RNA1 and RNA2 are almost identical (99%).

The protein encoded by *p22* has been shown to have RNA silencing suppression (RSS) activity (Kreuze et al., 2005) and was able to transiently increase viral titers of SPFMV after inoculation in sweetpotato (Cuellar et al., 2009). In *Nicotiana benthamiana*, p22 induces local necrosis when ectopically expressed in leaves by agroinfiltration and systemic necrosis when expressed from a viral vector (Kreuze et al., 2002). RNA silencing has a role in virus defense in plants (Baulcombe, 2004) and p22 may thus increase the severity of symptoms and virus interactions involving SPCSV. Indeed, co-infection of SPFMV with SPCSV isolates containing *p22* cause more severe symptoms (systemic necrosis) than co-infection of SPFMV with SPCSV isolates lacking *p22* in the indicator plant *Ipomoea setosa* (Cuellar et al., 2008). However, because isolates involved in these experiments have not been sequenced it cannot be excluded that this is caused by other factors than the *p22* gene.

Together with isolate Ug, isolate m2-47 is commonly used in experimental studies of viral synergisms in sweetpotato (Cuellar et al., 2009; Untiveros et al., 2007). In this study we determine the complete genome sequence of m2-47 isolate (Genbank ID: HQ291259 for RNA1 and HQ291260 for RNA2), confirming the absence of *p22* and also identifying other potentially important genomic differences. Through complementary expression in a transgenic plant we show evidence for a role of *p22* in the pathogenicity of SPCSV. We further show that *p22* is lacking from all available Peruvian and South American isolates and provide a model as to the mode of acquisition of *p22*.

SPCSV m2-47 was originally isolated from severely diseased sweetpotato plants growing in the Cañete valley of the southern coast of Lima by whitefly transmission on the indicator plant *I. setosa* (Gutierrez et al., 2003) and maintained by lateral grafting in an insect-proof greenhouse. Additional isolates used in this work (Table 1, samples 1–7) were isolated in the same way. Indicator plants were tested by NCM-ELISA for SPFMV, *Sweet potato virus G* (SPVG, Potyvirus), Sweet potato virus 2 (SPV2, Potyvirus), *Sweet potato latent virus* (SPLV, Potyvirus), *Sweet potato mild mottle virus* (SPMMV; *Ipomovirus*), *Sweet potato mild speckling virus* (SPMSV), *Sweet potato chlorotic fleck* (SPCFV, *Carlavirus*), C-6 virus, *Cucumber mosaic virus* (CMV, *Cucumovirus*) and Sweet potato caulimo-like virus (SPCV, *Caulimoviridae*) to confirm their absence. The plants were also checked to confirm the absence of begomoviruses by PCR (Li et al., 2004). Isolates 8–19 (Table 1; Untiveros et al., 2008) were maintained in sweetpotato and analyses were done on total RNA from these samples. Total RNA was isolated from 400 mg fresh *Ipomoea* leaves using Trizol (Invitrogen) following the manufacturer's instructions. RNA was resuspended in 250 μl sterile Milli-Q water (Sigma–Aldrich). The amount and quality of the RNA were checked using a spectrophotometer (Nanodrop, Thermo Scientific) and agarose gel electrophoresis. For isolate m2-47, RNA was lyophilized and sent to Fasteris Life Sciences SA (Plan-les-Ouates, Switzerland) for processing and high throughput sequencing (deep sequencing) on the Illumina Genome Analyzer II and short RNA viral sequences were assembled *in silico* using the programs Velvet and MAQ (http://maq.sourceforge.net) as previously described (Kreuze et al., 2009). Partial sequences of SPCSV isolates from Kisoro and Masaka were determined in a similar way, except that they were sent as a batch RNA prep with several other samples. To verify the integrity of the m2-47 genome assembled *in silico*, overlapping PCR products covering the span of the SPCSV genome were amplified using specifically designed primers and cloned using the pGEM-T easy cloning system (Promega) and *Escherichia coli* DH5α chemically competent cells. The sequence of two or more clones obtained from independent PCR reactions were confirmed by Sanger sequencing (Macrogen). Extension of these contigs, including sequences obtained by RT-PCR, was done using the program ContigExpress included in the Vector NTI package (Invitrogen). Transmembrane helices in p6 proteins were predicted with the TMHMM program (http://www.cbs.dtu.dk/services/TMHMM-2.0/) as previously described (Kreuze et al., 2002). Phylogenetic analyses were performed using the MEGA4 package (Kumar et al., 2008). To confirm the distinct 3′-UTR of RNA1 of m2-47 it was amplified using a custom RLM-RACE protocol as follows: ∼5 μg of RNA was incubated with 100 μM of Modban linker (5′rAppCTG TAG GCA CCA TCA AAT/3ddC/3′; IDT), 40 U of RNaseOUT (Invitrogen), 1x T4 RNA ligation buffer in a total volume of 20 μl and heated at 65 °C for 5 min. The reaction was then transferred to ice, 20 U of T4 RNA ligase (NEB) were added and incubated at 20 °C for 4 h. Then 7.5 μl of the RNA ligation mix was used for reverse transcription with the BanOne primer (5′-ATT GAT GGT GCC TAC AG-3′) and Superscript III reverse transcriptase (Invitrogen) according to the manufacturers recommendations at 50 °C for 55 min, followed by denaturation at 70 °C for 10 min and a treatment with 10 U of RNaseH (Invitrogen) at 37 °C for 20 min. Five microliters of the cDNA mix was then used for 35 cycles of PCR amplification using the BanOne and P7-F (5′-TTG ATG TGG CIC TAC TTT GGT-3′) primers with Platinum^®^ Taq polymerase (Invitrogen) according to the manufacturers recommendations. A specific fragment of the expected size of ∼310 bp was obtained, purified from gel, cloned into *E. coli* and sent for Sanger sequencing as described above. To determine the RNA1 and RNA2 3′-UTRs of remaining SPCSV isolates (Table 1), RNA from all samples were reverse-transcribed in a reaction mix (20 μl) containing 200 ng random hexamer primers, 10 mM dithiothreitol, 0.5 mM dNTPs, 20 U RNasin (Promega) and 400 U M-MLV reverse transcriptase (Invitrogen) at 37 °C for 1 h. The reaction was stopped by heating at 70 °C for 10 min and diluted fivefold with sterile water. Five microliters of the reaction (cDNA) was used as template for PCR using primers P7-F or SPCSV2-7242: (5′-ATT GAT GAG AAA TAA GCA CCG C-3′) and Crini-3UTR-R: (5′-TTT TTG AGI TTT TAI AAT ACA CAC-3′) flanking the *p22* insertion region. Samples lacking p22 produced a band of ∼250 bp. We confirmed the identity of the band of all isolates by restriction digestion (*not shown*) and of two EA and EA^p22^ isolates each by sequencing (Table 1). The 5′ region of RNA2 was also amplified and sequenced from seven isolates (Table 1) to confirm the presence of the *p6-SHP* (see below) using the primers SPCSV2-44f (5′-TAA GCT CGT ATC ATT GGT TGT CGT CA-3′) and SPCSV2-1046r (5′-GAC CTT CAT CGT ACC CCC GAC-3′). To study a possible role of p22 during SPCSV infection we used non-transgenic and *p22*-expressing (under control of the phloem specific RolC promoter) transgenic sweetpotato lines of cultivar ‘Huachano’ (Cuellar et al., 2009) and quantified the accumulation of m2-47 by triple antibody sandwich enzyme-linked immunosorbent assay (TAS-ELISA) (Karyeija et al., 2000).

The genome of SPCSV m2-47 consisted of approximately 8637nt for RNA1 and 8219nt for RNA2 and shared a total nt sequence identity of 98% with isolate Ug. The genome organization of m2-47 isolate differed from that of isolate Ug in three main ways (Fig. 1).

*The absence of a p22 gene in RNA1*: Northern blot results using a probe for RNase3 (*not shown*) and previous partial sequence characterization of SPCSV m2-47 indicating the absence of *p22* (Cuellar et al., 2008) is confirmed in this work. Results herein also indicate that *p22*-containing isolates are not common in other regions of South America (Table 1). In contrast, RNA samples collected from infected sweetpotatoes collected in the districts of Kisoro and Masaka in Uganda contained *p22*. Isolates of EA^p22^ have so far been reported only in the Mpigi province in Uganda (Cuellar et al., 2008; Kreuze et al., 2002). The presence of *p22* in isolates from other districts in Uganda further suggests that EA^p22^ is more common than previously known (Cuellar et al., 2008). Furthermore, the suspected presence in Kenya of isolates lacking *p22* (Hoyer et al., 1996a) is confirmed in this study (isolate Africa10, Table 1).

*An additional ORF encoding a small hydrophobic protein (SHP) upstream the hsp70h gene*: Isolates m2-47 and Ug contain a *p6* gene at the 5′ end of RNA2 showing 100% nt and aa identity. Although similar small ORFs found in the Closteroviridae encode SHP, no evidence of transmembrane domains in p6 have been detected (Kreuze et al., 2002; and results not shown), and instead the SHP is represented by the p7 on RNA1. However, in comparison to isolate Ug, SPCSV m2-47 contains an additional ORF of predicted 6 kDa (p6-SHP) with characteristic transmembrane helices predicted in its central region (Fig. 1). The p6-SHP showed no similarity in sequence to p6. A single nucleotide substitution (A575 → T) creates a stop codon interrupting a hypothetical *p6-SHP* in the corresponding region of isolate Ug. If not interrupted, this protein would show 88% aa identity to the p6-SHP of m2-47 and contain a similar predicted transmembrane helix region. Seven additional isolates analyzed in this study also contain *p6-SHP* in this region, suggesting that the lack of this ORF in SPCSV-Ug may be the result of a sequencing error. On the other hand the predicted first p6 ORF was not present in isolates Africa10 and Bitambi. The closteroviral SHP have a role in virus movement and have been found associated to membranes in the endoplasmic reticulum (Dolja et al., 2006). Duplication of SHPs in various positions of the genome is not uncommon among Closteroviridae, and they usually show little sequence similarity.

*3′-UTR of RNA1 and RNA2 differed significantly*: RNA1 and RNA2 of isolate m2-47 shows less than 82% nt identity at the 3′-UTR. In contrast, the 3′-UTRs of isolate Ug have a 99% nt sequence identity and are 99–99.5% identical to the 3′-UTR of m2-47 RNA2. Partial sequences of RNA 3′-UTRs of other SPCSV isolates lacking a *p22* ORF show they have a similar distinct RNA1-3′-UTR as m2-47 (Fig. 1B) when compared to SPCSV-Ug (Cuellar et al., 2008) (Fig. 1). This variability in sequence among virus isolates is not observed at the 5′-UTR.

All viruses encode at least one RSS protein (Li and Ding, 2006; Cañizares et al., 2008; Lu et al., 2004) and all SPCSV isolates contain the *RNase3* RSS (Cuellar et al., 2008). The low sequence conservation between the RSS genes of different viruses has been suggested as an indication of their relatively recent and independent acquisitions by viruses to counteract RNA silencing (Li and Ding, 2006). On the other hand, recombination-mediated gene gain has been suggested as a frequent phenomenon in the *Closteroviridae* (Dolja et al., 2006) explaining the incorporation of genes such as homologs of plant heat shock proteins (*hsp70h*) and *RNase III*-like genes with marginal aa sequence similarity to cellular RNase III enzymes (Dolja et al., 2006). The exceptional high similarity between the 3′-UTR of RNA1 and RNA2 of SPCSV-Ug, not observed in other criniviruses, also suggest recombination as a mechanism shaping the genome organization of SPCSV. Interestingly the high similarity in the 3′-UTRs of RNA1 and RNA2 seems common among EA^p22^ isolates, but is not found in EA or in the quite distant WA strain isolates (Cuellar et al., 2008; Fig. 1B). We suggest that the incorporation of *p22* in RNA1 may be related to the high sequence identity between the 3′-UTR of RNA2 and RNA1 observed in EA^p22^ isolates: *i.e. p22* may have been acquired after its own 3′-UTR recombined with the 3′-UTR of SPCSV RNA2 (including the last 5 codons of p28; Fig. 1B) which enabled it to be recognized by the viral polymerase, thus perhaps facilitating its subsequent incorporation into RNA1 (Fig. 1). The alternative explanation, that *p22* was ancestrally present and subsequently lost seems less likely as an almost identical excision event and acquisition of divergent 3′-UTR would need to have occurred in the distantly related EA and WA strains of SPCSV.

Only limited phenotypic differences between single-infection with EA and EA^p22^ isolates can be observed in sweetpotato (Cuellar et al., 2008). In addition a critical role for p22 in development of SPVD has been discarded (Cuellar et al., 2008, 2009). Accordingly, isolates collected in this study and co-infected with SPFMV showed symptoms of SPVD regardless of the absence of *p22* (Table 1). To study the role of *p22* on the biology of SPCSV we used transgenic sweetpotato expressing *p22* under a phloem specific promoter (Cuellar et al., 2009), thus delivering the protein *in trans* and avoiding the effects of isolate sequence variability on virus accumulation. Results from TAS-ELISA experiments indicate that although differences in SPCSV accumulation were only significant in one event (*p* < 0.05, Fig. 2) the average titers of SPCSV-m2-47 in *p22* transgenic sweetpotato plants were always higher than in non-transgenic plants. This is consistent with previous observations where we show that isolate Ug accumulate at higher levels in sweetpotato (cultivar “Tanzania”) as compared to other EA isolates (Cuellar et al., 2008), and suggests the activity of p22 alone is sufficient to explain this difference.

Given that isolates containing *p22* have not been found in regions outside East Africa (Cuellar et al., 2008) including the Americas (*this work*) and considering reports on East Africa as a region hosting a high variability of sweetpotato viruses and virus strains, including SPCSV (Alicai et al., 1999; Gibson et al., 1998; Tugume et al., 2010; Tairo et al., 2005) it is likely that the acquisition event of *p22* may have occurred rather recently in East Africa (specifically Uganda), a region where it is possible to find both EA and EA^p22^ isolates (Cuellar et al., 2008; Hoyer et al., 1996a; *this study*) and where breeding for virus-resistance in sweetpotato has been an intense activity (Aritua et al., 1999; Mwanga et al., 2001). Strong selection pressure exerted by highly resistant sweetpotato grown in the region may thus have lead to evolution and subsequent spread of EA^p22^. Although the origin of *p22* remains unknown, plausible possibilities are the host genome or an as yet unidentified co-infecting (crini-)virus.

## Figures and Tables

**Fig. 1:**
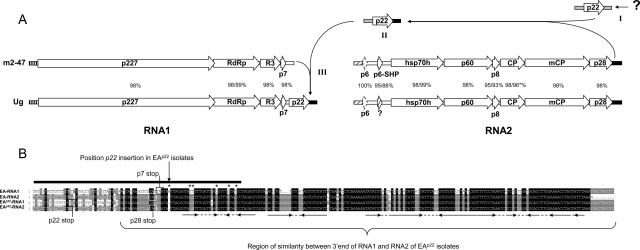
(A) Schematic representation and comparison of *Sweet potato chlorotic stunt virus* genome organization of isolates m2-47 and Ug. Percentage values represent the identity at nucleotide level for each ORF; proteins identities are given behind the slash if different from nucleotide. * includes 3nt/1aa deletion in the CP of isolate m2-47. Different patterns of shading at the terminal parts of the genomes of both isolates represent differences in sequence conservation at these regions. There is a second ORF encoding a putative protein with transmembrane domain (p6-SHP) in m2-47 and the six other isolates (Table 1) sequenced in this region, the percentages shown correspond to its comparison with the same region in isolate Ug, except that a single nucleotide change (A575 → T) interrupts the *p6-SHP* in Ug (indicated by a question mark). The *p6* is absent from isolates (Africa10 and Bitambi) and is indicated by dotted lines. Arrows show a suggested route for *p22* acquisition, with roman numerals indicating a suggested sequence of steps giving origin to *p22* in isolate Ug. A question mark indicates the unknown source of *p22*. (B) Alignment of the 3′-UTRs of EA and EA^p22^ isolates characterized in this study (Table 1) highlighting the dissimilarity in this region. The bold line above the alignment on the left indicates the region of RNA1-3′-UTR sequenced previously for other isolates lacking *p22* (Cuellar et al., 2008), and nucleotides conserved between those and m2-47 are indicated with an asterisk. Arrows below the alignment indicate conserved secondary structure of the 3′-UTR found in all criniviruses; stop codons of the 3′ terminal ORFs are indicated by boxes. The last 10 nts of m2-47 RNA2 were not sequenced.

**Fig. 2:**
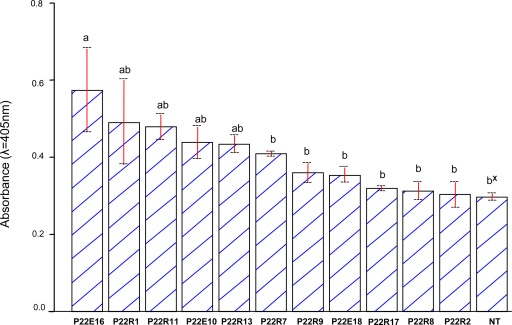
Accumulation of *Sweet potato chlorotic stunt virus* (SPCSV) isolate m2-47 in non-transgenic and transgenic sweetpotato expressing the *p22* gene of SPCSV. Virus titers were measured by triple antibody sandwich enzyme-linked immunosorbent assay (Karyeija et al., 2000) in leaves of the middle part (average of the 5th and 6th leaf) of three plants 6 weeks after infection. Different letters over each bar indicate significant differences according to the test of High Significance Difference – HSD, *α* = 0.05. NT = non-transgenic sweetpotato.

**Table 1:** *Sweet potato chlorotic stunt virus* isolates used in this study and sequence accession numbers. Strain was determined by serology using antiserum MAb mix 1 and mix 2 (Gutierrez et al., 2003) for isolates 1–19 and by sequence analysis of *hsp70h* for isolates 20 and 21. Presence or absence of *p22* was determined by PCR-RFLP as described in materials and methods and confirmed by sequencing in selected cases.

SPCSV Isolate	Origin	Strain	*p22*	RNA1 3′-UTR	RNA2 3′-UTR	RNA2 5′-UTR
(1) m2-47	Cañete, Peru	EA	NO	HQ291259	HQ291260	HQ291260
(2) Set2	San Ramon, Peru	EA	NO			
(3) Set4	San Ramon, Peru	EA	NO			
(4) Pelotas	Pelotas, Brazil	WA	NO			
(5) EL	El Indio, Argentina	WA	NO			HQ847524
(6) Arg	Cordoba, Argentina	WA	NO	HQ847529	HQ847534	
(7) Africa10	Kenya	EA	NO			HQ847523
(8) SR	San Ramon, Peru	EA	NO			
(9) Fe	Ferreñafe, Peru	EA	NO			
(10) Fio	Cañete, Peru	EA	NO	HQ847528	HQ847533	HQ847522
(11) KmtMil	Cañete, Peru	EA	NO			
(11) m2-41	Cañete, Peru	EA	NO			
(12) m2-44	Cañete, Peru	EA	NO			
(13) m2-63	Cañete, Peru	EA	NO			
(14) C14	Cañete, Peru	EA	NO			
(15) C18	Cañete, Peru	EA	NO			
(16) C21	Cañete, Peru	EA	NO			
(17) Hua	Huaral, Peru	EA	NO			HQ847520
(18) Chi4	Chimbote, Peru	EA	NO	HQ847527	HQ847532	HQ847521
(19) Chi2	Chimbote, Peru	EA	NO			
(20) KSR675	Kisoro, Uganda	EA	YES	HQ847530	HQ847535	HQ847525
(21) Bitambi	Masaka, Uganda	EA	YES	HQ847531	HQ847536	HQ847526

## References

[bib0005] Alicai T., Fenby N.S., Gibson R.W., Adipala E., Vetten H.J., Foster G.D., Seal S.E. (1999). Occurrence of two serotypes of sweet potato chlorotic stunt virus in East Africa and their associated differences in coat protein and hsp70 homologue gene sequences. Plant Pathol..

[bib0010] Aritua V., Legg J.P., Smit N.E.J.M., Gibson R.W. (1999). Effect of local inoculum on the spread of sweet potato virus disease: limited infection of susceptible cultivars following widespread cultivation of a resistant sweet potato cultivar. Plant Pathol..

[bib0015] Baulcombe D.C. (2004). RNA silencing in plants. Nature.

[bib0020] Cañizares M.C., Navas-Castillo J., Moriones E. (2008). Multiple suppressors of RNA silencing encoded by both genomic RNAs of the crinivirus, Tomato chlorosis virus. Virology.

[bib0025] Cuellar W.J., Kreuze J.F., Rajamäki M.L., Cruzado K.R., Untiveros M., Valkonen J.P.T. (2009). Elimination of antiviral defense by viral RNase III. Proc. Natl. Acad. Sci. U. S. A..

[bib0030] Cuellar W.J., Tairo F., Kreuze J.F., Valkonen J.P.T. (2008). Analysis of gene content in *Sweet potato chlorotic stunt virus* RNA1 reveals the presence of p22 RNA silencing suppressor in only few isolates: implications to viral evolution and synergism. J. Gen. Virol..

[bib0035] Di Feo L.D., Nome S.F., Biderbost E., Fuentes S., Salazar L.F. (2000). Etiology of sweet potato chlorotic dwarf disease in Argentina. Plant Dis..

[bib0040] Dolja V.V., Kreuze J.F., Valkonen J.P.T. (2006). Comparative and functional genomics of closteroviruses. Virus Res..

[bib0045] Gibson R.W., Mpembe I., Alizai T., Carey E.E., Mwanga R.O.M., Seal S.K., Vetten H.F. (1998). Symptoms, aetiology and serological analysis of sweet potato virus disease in Uganda. Plant Pathol..

[bib0050] Gutierrez D.L., Fuentes S., Salazar L.F. (2003). Sweetpotato virus disease (SPVD): distribution, incidence, and effect on sweetpotato yield in Peru. Plant Dis..

[bib0055] Hoyer U., Jelkmann W., Maiss E., Vetten H.J. (1996). Sweet potato sunken vein virus (SPSVV): another bipartite closterovirus transmitted by Bemisia tabaci. Phytoparasitica.

[bib0060] Hoyer U., Maiss E., Jelkmann W., Lesemann D.-E., Vetten H.J. (1996). Identification of the coat protein gene of a sweet potato sunken vein closterovirus isolate from Kenya and evidence for a serological relationship among geographically diverse closterovirus isolates from sweetpotato. Phytopathology.

[bib0065] Karyeija R.F., Kreuze J.F., Gibson R.W., Valkonen J.P.T. (2000). Synergistic interaction of a potyvirus and a phloem-limited crinivirus in sweetpotato plants. Virology.

[bib0070] Kokkinos C.D., Clark C.A. (2006). Interactions among sweet potato chlorotic stunt virus and different potyviruses and potyvirus strains infecting sweetpotato in the United States. Plant Dis..

[bib0075] Kreuze J.F., Perez A., Untiveros M., Quispe D., Fuentes S., Barker I., Simon R. (2009). Complete viral genome sequence and discovery of novel viruses by deep sequencing of small RNAs: a generic method for diagnosis, discovery and sequencing of viruses. Virology.

[bib0080] Kreuze J.F., Savenkov E.I., Cuellar W.J., Li X., Valkonen J.P.T. (2005). Viral class 1 RNase III involved in suppression of RNA silencing. J. Virol..

[bib0085] Kreuze J.F., Savenkov E.I., Valkonen J.P.T. (2002). Complete sequence and analyses of the subgenomic RNAs of *Sweet Potato Chlorotic Stunt Virus* reveal several new features for the genus Crinivirus. J. Virol..

[bib0090] Kumar S., Nei M., Dudley J., Tamura K. (2008). MEGA: a biologist-centric software for evolutionary analysis of DNA and protein sequences. Briefings BioInformatics.

[bib0095] Li F., Ding S.W. (2006). Virus counterdefense: diverse strategies for evading the RNA-silencing immunity. Ann. Rev. Microbiol..

[bib0100] Li R., Salih S., Hurtt S. (2004). Detection of geminiviruses in sweetpotato by polymerase chain reaction. Plant Dis..

[bib0105] Lu R., Folimonov A., Shintaku M., Li W.X., Falk B.W., Dawson W.O., Ding S.W. (2004). Three distinct suppressors of RNA silencing encoded by a 20-kb viral RNA genome. Proc. Natl. Acad. Sci. U. S. A..

[bib0110] Milgram M., Cohen J., Loebenstein G. (1996). Effects of Sweet potato feathery mottle virus and sweet potato sunken vein virus on sweet potato yields and rates of reinfection of virus-free planting material in Israel. Phytoparasitica.

[bib0115] Mukasa S.B., Rubaihayo P.R., Valkonen J.P.T. (2006). Interactions between a crinivirus, an ipomovirus and a potyvirus in co-infected sweetpotato plants. Plant Pathol..

[bib0120] Mwanga R.O.M., Odongo B., Ocitti-p’Obwoja C.N., Gibson R.W., Smit N.E.J.M., Carey E.E. (2001). Release of five sweetpotato cultivars in Uganda. HortScience.

[bib0125] Tairo F., Mukasa S.B., Jones R.A., Kullaya A., Rubaihayo P.R., Valkonen J.P.T. (2005). Unraveling the genetic diversity of the three main viruses involved in sweet potato virus disease (SPVD), and its practical implications. Mol. Plant Pathol..

[bib0130] Tugume A.K., Cuellar W.J., Mukasa S.B., Valkonen J.P.T. (2010). Molecular genetic analysis of virus isolates from wild and cultivated plants demonstrates that East Africa is a hotspot for the evolution and diversification of Sweet potato feathery mottle virus. Mol. Ecol..

[bib0135] Untiveros M., Fuentes S., Kreuze J.F. (2008). Molecular variability of sweet potato feathery mottle virus and other potyviruses infecting sweet potato in Peru. Arch. Virol..

[bib0140] Untiveros M., Fuentes S., Salazar L.F. (2007). Synergistic interaction of sweet potato chlorotic stunt virus (*Crinivirus*) with carla-, cucumo-, ipomo-, and potyviruses infecting sweet potato. Plant Dis..

[bib0145] Valverde R., Moreira M.A. (2004). Identificación de virus en el cultivo de camote (*Ipomoea batatas* L.) en Costa Rica. Agronomía Mesoamericana.

[bib0150] Vetten H.J., Hoyer U., Maiss E., Lesemann D.-E., Jelkmann W. (1996). Serologicla detection and discrimination of geographically diverse isolates of sweet potato sunken vein closterovirus. Phytopathology.

